# Mechanisms of MTA3 in cancer and related diseases and its clinical applications

**DOI:** 10.3389/fonc.2025.1731799

**Published:** 2026-01-09

**Authors:** Yan Tang, Xiao-Jiao Li, Hui Ao, Qian-Guo Liu, Xiao-Fu Zheng, Chang-Li Liao, Jun Li, Yong-Kang Wu

**Affiliations:** 1 Department of Ultrasound Medicine, West China Hospital Sichuan University Jintang Hospital. Jintang First People’s Hospital, Chengdu, China; 2 Department of Respiratory and Critical Care Medicine, West China Hospital Sichuan University Jintang Hospital. Jintang First People’s Hospital, Chengdu, China; 3 Department of Laboratory Medicine, West China Hospital, Sichuan University, Chengdu, China; 4 West China Hospital Sichuan University Jintang Hospital, Jintang First People’s Hospital, Chengdu, China

**Keywords:** cancer, metastasis-associated protein, MTA3, NuRD complex, oncogene

## Abstract

Metastasis-associated protein 3 (MTA3), a key member of the MTA family, is an integral component of the nucleosome remodeling and deacetylase complex, with widespread expression across diverse human tissues and organs. By modulating epigenetic modifications, MTA3 is instrumental in regulating vital physiological processes, including cell differentiation, apoptosis, and metabolism. It plays a crucial role in maintaining normal tissue homeostasis and exerts a significant regulatory influence on pathological conditions, notably cancer and other diseases. This review presents a comprehensive evaluation of the molecular structural characteristics and biological functions of MTA3, providing a detailed overview of its mechanistic role in tumorigenesis and disease progression. Its unique tissue-specific expression patterns and dual functional roles as an oncogene or tumor suppressor, depending on the cellular and disease context, are highlighted. Moreover, by integrating recent research advancements, the feasibility and potential clinical translational value of using MTA3 as a diagnostic and prognostic biomarker, as well as a therapeutic target in developing novel disease intervention strategies, are evaluated in this review. This study aimed to establish a robust theoretical foundation and provide novel research perspectives to support future endeavors focused on improving patient outcomes by precisely modulating MTA3 activity.

## 1 Introduction

The metastasis-associated protein (MTA) family is a primary focus of cancer research, with its members playing unique and frequently synergistic roles in regulating tumorigenesis, disease progression, recurrence, angiogenesis, and metastasis (1). MTA3, a principal member of this family, plays a crucial role in cancer regulatory networks by directly and indirectly modulating key oncogenic factors, including the transcriptional repressor Snail, E-cadherin, signal transducer and activator of transcription (STAT) proteins, and estrogen receptors (2, 3).

Research on the MTA family commenced in 1994 with the identification and functional characterization of MTA1 (4). MTA2 was discovered in 1999 (5), and the cloning and characterization of murine MTA3 in 2001 sparked comprehensive investigations into all family members (6). MTA1, MTA2, and MTA3 exhibit different molecular weights. MTA3 shares approximately 80% amino acid sequence homology with MTA1, indicating potential partial functional redundancy; however, its unique structural domains confer specialized and divergent biological functions. Functional studies have demonstrated that, unlike MTA1 and MTA2, which predominantly facilitate tumor progression, MTA3 was initially reported to suppress cancer cell invasion and metastasis (7, 8). Subsequent studies revealed a more intricate role for MTA3 in tumorigenesis, revealing context-dependent dual functionality—either promoting or inhibiting cancer progression—depending on tumor type, disease stage, and the microenvironment (2).

Considering the key regulatory function of MTA3 in cancer and other human diseases, as well as the lack of comprehensive reviews summarizing its mechanistic actions and clinical translational potential, this article is dedicated to MTA3. It systematically elucidates the molecular mechanisms underlying MTA3’s involvement in various diseases and evaluates its prospective applications in diagnosis and therapeutic development. This study establishes a theoretical foundation for future mechanistic investigations and clinical interventions.

### 1.1 Literature search

Two independent reviewers conducted a comprehensive search across multiple electronic databases, including PubMed, Embase, Web of Science, and Cochrane, to identify relevant studies published after 2001. The search strategy employed the following terms: “MTA3,” “Metastasis-associated protein 3,” “MTA3 gene,” “NuRD complex,” “chromatin remodeling,” “transcriptional corepressor,” and “epigenetic regulation.” The search was restricted to literature published in English. Reference management was performed using EndNote 20 software. In instances of disagreement between the reviewers, a consensus was achieved through consultation with a third reviewer.

### 1.2 Inclusion and exclusion criteria for literature

Selection inclusion criteria: (1) The research must directly address the expression, regulation, function, molecular mechanisms, or role of MTA3 (or its encoding gene) in the context of diseases. (2) Acceptable research models encompass, but are not limited to, *in vitro* cell line models, *in vivo* animal models (e.g., mice, rats), and human tissue samples (e.g., tumor specimens, blood samples). (3) Eligible research types include original research articles, review articles (utilized for background information and identification of primary literature), case reports (specifically if they pertain to MTA3 mutations or distinct clinical manifestations), and conference abstracts (solely for acquiring the latest advancements in early translational research). (4) The study must explicitly investigate the association between MTA3 and cancer or other relevant diseases (such as developmental or metabolic diseases, if identified during the search process).

Exclusion criteria: (1) Studies that do not specifically address MTA3 or merely refer to it as a non-specific element of the NuRD complex. (2) Literature for which the full text is inaccessible, such as abstracts lacking comprehensive information. (3) Redundant publications, with only the earliest or most comprehensive version being retained. (4) Non-research articles, including editorials and reviews (unless they provide significant expert opinions or valuable insights into the field), as well as news reports, among others.

## 2 Biological characteristics of MTA3

### 2.1 Gene localization and molecular structure of MTA3

MTA3 is an integral component of the nucleosome remodeling and deacetylase (NuRD) complex. The gene encoding MTA3 is located on human chromosome 2p21 and contains 20 exons. Its promoter region contains multiple transcription factor-binding sites, including those for Sp1, AP-1, and estrogen receptor (ER), suggesting a complex and multifactorial mechanism of transcriptional regulation (1, 6, 9). The MTA3 protein comprises 515 amino acids with a molecular weight of approximately 60 kDa, and is highly conserved across species, sharing about 80% sequence similarity with MTA1 and MTA2 (6).

**Figure 1:**
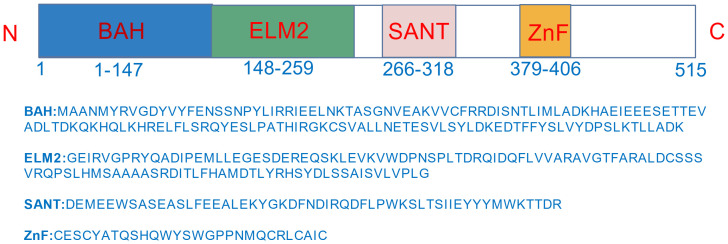
Structure of MTA3 protein. A schematic representation of the MTA3 protein structure, illustrating its composition of four functional domains: BAH, ELM2, SANT, and ZNF, which collectively constitute its complete architecture.

MTA3 contains several functional domains (**Figure 1**): BAH domain mediates protein–protein interactions (PPIs) and gene regulation; SANT domain serves as a transcription factor scaffold involved in chromatin remodeling; ELM2 domain interacts with histone deacetylases, thereby influencing transcriptional activity and embryonic development. Additionally, GATA-type zinc finger domain (residues 379–406) is predicted to confer DNA-binding capability and contribute to tissue-specific functions (1, 6, 9, 10). MTA3 also contains an SH3-binding domain, acidic regions, and a nuclear localization signal, underscoring its critical role in chromatin remodeling and transcriptional regulation (6, 9).

At least seven alternative splicing variants of MTA3 have been identified to date. Notably, the shorter isoform, MTA3s, demonstrated elevated expression levels at the mRNA and protein levels. Variants such as MTA3L exhibit structural differences in their C-terminal regions, potentially affecting their protein interaction profiles and functional characteristics (9). MTA3 expression is modulated by various factors, including transcription factors (p53 and Kaiso), growth factors and their receptors (VEGF, EGFR, Heregulin β1, and ErbB2), and microRNAs (miRNAs; miR-661, miR-30c, and miR-125a-3p) (11–13). These elements form a complex regulatory network that supports MTA3’s extensive and context-dependent roles in physiological and pathological processes.

### 2.2 Functional expression of MTA3 in normal tissues

MTA3 contributes to the development and homeostasis of multiple tissues and organs through epigenetic regulatory mechanisms. It is critical in maintaining stem cell pluripotency, hematopoietic system development, immune cell differentiation, and reproductive system function.

In embryonic stem cells, MTA3 cooperates with MTA2 to maintain pluripotency. MTA3 knockdown induces mesodermal differentiation in a manner dependent on the activity of dual-specificity tyrosine-phosphorylation-regulated kinase DYRK4, an effect that can be reversed by the inhibitor ID8 (14). Furthermore, S-nitrosylation of MTA3 decreases the activity of the NuRD complex, thereby reducing the reprogramming efficiency of induced pluripotent stem cells (15).

In the hematopoietic system, MTA3 functions as a key regulator of primitive hematopoiesis. MTA3 knockdown in zebrafish models led to the absence of the primitive hematopoietic lineage and aberrant angiogenesis, whereas MTA3 overexpression improved the expression of hematopoietic transcription factors gata1 and hbbe3 (16). MTA3 cooperates with other components of the NuRD complex (MBD3 and HDAC1) to regulate the expression of early hematopoietic genes scl and lmo2, a process that depends on its histone deacetylase activity (17).

Within the immune system, MTA3 is co-expressed with the transcriptional repressor BCL-6 in germinal center B cells. It mediates BCL-6-dependent transcriptional repression *via* the NuRD complex, thereby preventing premature differentiation of B cells into plasma cells. MTA3 depletion mediated by RNA interference disrupts plasma cell-specific transcriptional programs and induces re-expression of cell surface markers (18). Recent studies have indicated that MTA3 indirectly modulates humoral immunity by regulating follicular helper T cell (Tfh) differentiation (19).

MTA3 exhibits diverse functions in reproductive and endocrine-related tissues. In the ovary, MTA3 is highly expressed in granulosa cells, where it regulates G2/M phase progression by interacting with the NuRD complex and desmin-containing structures, thereby modulating cell proliferation (20). In placental tissue, MTA3 is induced under hypoxic conditions and upregulates HIF1α expression, contributing to trophoblast differentiation and functional regulation (21). Aberrant MTA3 expression is associated with the pathogenesis of preeclampsia (22). During mammary gland development, MTA3 suppresses ductal branching by inhibiting the Wnt4 signaling pathway, indicating its potential role in regulating stem cell fate decisions (23). Moreover, MTA3 colocalizes with the chromatin remodeling factor CHD5 in brain tissue, implicating it in neural development and tumor suppression (6, 24).

In summary, MTA3 integrates epigenetic regulation with cellular signaling networks to mediate tissue-specific functions in various physiological contexts. Its mechanisms involve post-translational modifications, dynamic complex assembly, and selective repression of target genes, positioning MTA3 as a critical molecular node in developmental biology and a promising candidate for therapeutic intervention in diseases.

### 2.3 The main signaling pathways of MTA3 in related diseases

**Figure 2:**
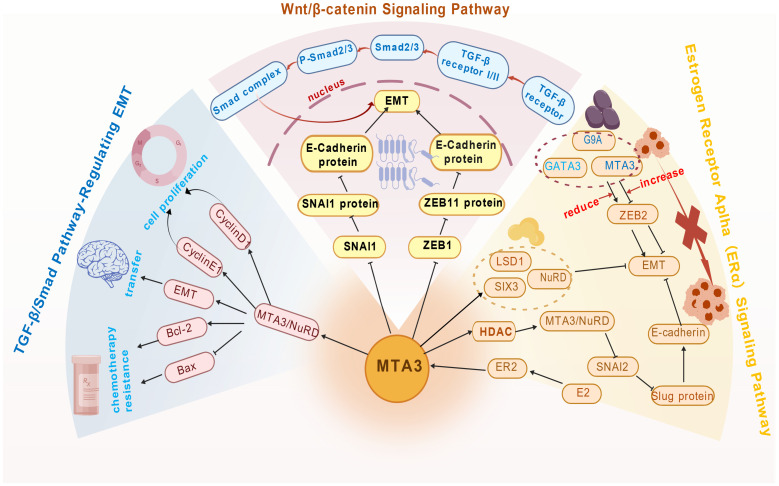
Relevant signaling pathways of MTA3’s function. A schematic representation of the principal signaling pathways associated with the MTA3 protein, illustrating its regulatory influence on critical biological processes, including the cell cycle, apoptosis, and migration, through interactions with multiple pathways.

MTA3 regulates gene expression through epigenetic mechanisms and is implicated in both neoplastic and non-neoplastic diseases (**Figure 2**). In neoplastic conditions, such as breast cancer, MTA3 primarily acts as a tumor suppressor. Its core mechanism involves the MTA3/ERα/Snail signaling axis. Estrogen-activated ERα recruits the MTA3/NuRD complex to the Snail promoter region, where transcription is repressed *via* histone deacetylation. Suppression of Snail expression helps maintain the epithelial phenotype, inhibits epithelial-mesenchymal transition (EMT), and reduces tumor invasion and metastasis (10, 25). Moreover, MTA3 antagonizes the Wnt/β-catenin and TGF-β signaling pathways by promoting NuRD-mediated repression of downstream oncogenes, including c-Myc, thereby inhibiting cell proliferation and EMT (26).

In non-neoplastic diseases, MTA3 exerts regulatory effects by modulating inflammatory and fibrotic processes. In models of acute liver injury, MTA3 mitigates inflammation by suppressing NF-κB transcriptional activity and downregulating pro-inflammatory cytokines, including TNF-α and IL-6 (27). Similarly, MTA3 delays the progression of organ fibrosis by inhibiting transcriptional outputs of the TGF-β/Smad signaling pathway (28).

In summary, as a key adaptor within the NuRD complex, MTA3 precisely regulates multiple key signaling pathways by directing the recruitment of histone-modifying complexes to specific gene promoters.Its potential holds significant exploratory value and is anticipated to offer novel scientific foundations for the analysis of mechanisms and the development of therapeutic intervention strategies for related diseases, including breast cancer, hematological disorders, and various tumors.

## 3 MTA3 expression in oncological diseases

### 3.1 Breast cancer

Breast cancer, a malignant proliferative disorder of mammary epithelial tissue, involves dysregulation of multiple molecular pathways during its initiation and progression. MTA3 exhibits significant tumor-suppressive effects in breast cancer. Its expression is regulated by the transcription factors SP1 and ER and depends on ERα and estradiol signaling, positioning MTA3 as a key downstream effector in the ER signaling pathway (69, 70). At the molecular level, MTA3 directly represses transcription of Snail, a central regulator of EMT, thereby maintaining expression of the epithelial marker E-cadherin and inhibiting tumor cell invasion and metastasis (10, 25). Moreover, MTA3 is involved in multiple transcriptional repression complexes. For example, SIX3/LSD1/NuRD(MTA3) complex suppresses breast cancer initiation and metastatic progression (29); GATA3/G9A/NuRD(MTA3) complex inhibits tumor invasion in both *in vitro* and *in vivo* models, and its downregulation leads to ZEB2 upregulation and promotes malignant progression (13).

**Figure 3:**
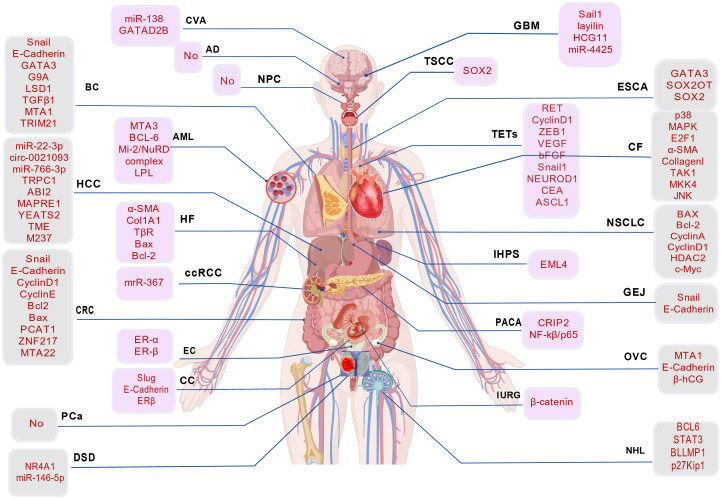
Abnormal expression of MTA3 in neoplastic and non-neoplastic diseases. A schematic diagram illustrating the principal signaling pathways associated with the MTA3 protein, highlighting its regulatory role in critical biological processes, including the cell cycle, apoptosis, and migration, through various pathway interactions. CVA, Cerebrovascular Accident; AD, Alzheimer’s disease; GBM, Glioblastoma; NPC, Nasopharyngeal carcinoma; TSCC, Lip squamous cell carcinoma; ESCA, Esophageal cancer; TETs, Thymic epithelial tumor; MF, Cardiac fibrosis; BC, Breast cancer; NSCLC, Non-small cell lung cancer; IHPS, Infantile hypertrophic pyloric stenosis; GEL, Adenocarcinoma of the gastroesophageal junction; HCC, Hepatocellular carcinoma; HF, Hepatic fibrosis; ccRCC, Clear Cell Renal Cell Carcinoma; PDAC, Pancreatic ductal adenocarcinoma; CRC, Colorectal cancer; EC, Endometrial cancer; CC, Choriocarcinoma; OVC, ovarian cancer; IURG, Intrauterine Growth Retardation; Pca, Prostate cancer; DSD, Disorders in testicular steroidogenesis; NHL, Non-Hodgkin Lymphoma; AML, Acute Myelocytic Leukemia.

MTA3 expression is significantly associated with breast cancer prognosis. In ERα-positive tumors, high MTA3 expression is positively correlated with E-cadherin levels, indicating a favorable clinical outcome (12, 30). Loss of MTA3 expression is frequently observed in advanced-stage cancers and is accompanied by reduced E-cadherin expression and aberrant β-catenin localization (11). Furthermore, therapeutic agents, including the histone deacetylase inhibitor LBH589 and the natural compound β-elemene, modulate cancer cell migration and invasion by regulating the MTA3–Snail–E-cadherin axis (12, 31). MTA3 also interacts with proteins such as MTA1 and TRIM21 to regulate breast cancer stemness and EMT, and this regulatory equilibrium is modulated by estrogen signaling (32) (see **Table 1**, **Figure 3**).

**Table 1:** Expression profile of MTA3 in neoplastic diseases.

Cancer	Mechanism pathway	Interacting genes or proteins	Expression	Functions	References
Breast cancer	1. EMT Regulation (Snail/E-Cadherin Axis) 2. LSD1/MTA3/NuRD 3. MTA1-MTA3/TRIM21	Snail,E-Cadherin,GATA3,G9A,LSD1,TGFβ1,MTA1,TRIM21	Downregulation	Cancer stemness, EMT, cell cycle progression	(10, 13, 29, 30, 32)
NSCLC	1. Cell Cycle Regulation (Cyclin A/Cyclin D1/p-Rb) 2. The miR-495-MTA3 axis regulates epithelial-mesenchymal transition (EMT) 3. The MTA3-HDAC2 interaction upregulates c-Myc expression	BAX,Cleaved-Caspase-3,p-PARP,Bcl-2,CyclinA,CyclinD1,p-Rb,miR-495,HDAC2,c-Myc	Overexpression	Promote the proliferation, migration and invasion of cancer cells;	(33–37)
HCC	1.The miR-22-3p–MTA3 Axis 2.The circ_0021093/miR-766-3p/MTA3 axis 3.The TRPC1-MTA3 signaling pathway (upregulating MTA3)	miR-22-3p,circ_0021093,miR-766-3p,TRPC1,ABI2,MAPRE1,YEATS2,TMEM237	Nuclear Overexpression with cytoplasmic Downregulation	Promotes cancer cell proliferation, invasion, and metastasis;	(38–41)
Glioma	1.The layilin-MTA3-SNAI1 axis regulates epithelial-mesenchymal transition (EMT) 2.The HCG11-miR-4425-MTA3 Axis	Sail1,layilin,HCG11,miR-4425	Downregulation	Inhibit tumor growth;	(42–44)
NPC	Race-specific regulation	/	Overexpression	Related to prognosis, recurrence, and metastasis	(45, 46)
TSCC	The MTA3-SOX2 axis regulates the characteristics of cancer stem cells	SOX2	Downregulation	Inhibiting the characteristics of cancer stem cells and tumor growth	(47–49)
Esophageal cancer	SOX2OT/SOX2 axis	GATA3,SOX2OT, SOX2	Downregulation	Inhibition of cancer cell proliferation and metastasis	(50, 51)
GEJ	EMT regulatory mechanism (Snail/E-cadherin axis)	Snail, E-Cadherin	Downregulation	Inhibition of metastasis	(51–53)
Colorectal cancer	1. Activation of Wnt Signaling Pathway 2. Regulation of EMT (Snail1/E-cadherin Axis) 3. Cell Cycle Regulation (Cyclin D1/Cyclin E) 4. Apoptosis regulation (Bcl2/Bax)	nail1,E-Cadherin,Cyclin D1,Cyclin E,Bcl2,Bax,PCAT1,ZNF217,MTA2	Overexpression	Promotes the proliferation, invasion, and migration of cancer cells; enhances resistance to 5-fluorouracil.	(26, 54, 55)
ccRCC	miR-367-MTA3 axis	miR-367	Downregulation	Overexpression of MTA3 can reverse the effects of miR-367 on cell proliferation, migration an invasion	(56)
Prostate cancer	MTA3 pathway enrichment	No	Overexpression	Related to tumor occurrence	(57)
Endometrial cancer	ER-α/ER-β-MTA3 axis	ER-α,ER-β	Downregulation	Surgical staging, lymph node metastasis, and poor prognosis related	(58, 59)
Choriocarcinoma	ERβ-MTA3/Slug/E Cadherin Axis	Slug, E - Cadherin, ERβ	Overexpression	Promote cell migration and angiogenesis	(60, 61)
Ovarian cancer	MTA1-MTA3-E - Cadherin Axis	MTA1,E-Cadherin,β-hCG	Downregulation	Inhibits metastasis; low expression of MTA3 promotes disease onset and invasive progression	(59, 62)
TETs	1.MTA3-RET pathway 2.EMT regulation (Snail1/ZEB1)	RET, Cyclin D1, c-Myc, Bcl-2, Survivin, ASCL1, NEUROD1, CEA, Snail1, ZEB1, VEGF, bFGF	Bidirectionality	high expression maintains the neuroendocrine phenotype, low expression promotes EMT	(63, 64)
Leukemia	MTA3 signaling pathway; cooperates with Mi-2/NuRD complex and BCL-6 to regulate B cell differentiation and dedifferentiation	MTA3, BCL-6, Mi-2/NuRD complex, LPL	Downregulation	Differentiation	(18, 65)
Lymphoma	1.The interaction between MTA3 and BCL6 regulates transcription 2. BCL6-MTA3 regulates the functions of B cells in the germinal center	BCL6,STAT3, BLIMP1, p27kip1	Downregulation	Differentiation, Proliferation, angiogenesis	(66–68)

Currently, the majority of research concerning MTA3 in breast cancer remains at the preclinical exploration and early translational stages. Future investigations should prioritize elucidating the regulatory mechanisms of MTA3 expression and its functional characteristics in estrogen receptor-negative breast cancer, particularly in triple-negative breast cancer. Employing preclinical research methodologies, such as cell-based assays and animal models, could facilitate the initial clarification of MTA3’s specific roles across various molecular subtypes in epithelial-mesenchymal transition (EMT), invasion, metastasis, and the maintenance of stemness. Concurrently, a range of technical approaches could be utilized to dissect the composition and dynamic regulatory networks of MTA3-containing complexes. Through preclinical studies and preliminary translational research, the potential of MTA3 as a prognostic biomarker or therapeutic target could be cautiously assessed, thereby providing a foundational theoretical basis and guiding future research directions for the precision treatment of breast cancer. Nonetheless, to enable its widespread clinical application, extensive, in-depth, and rigorous research is essential for validation.

### 3.2 Non-small cell lung cancer

Non-small cell lung cancer (NSCLC) is the most prevalent form of lung cancer, accounting for approximately 85% of all cases. Its development and progression are linked to multiple molecular aberrations (71). In NSCLC, MTA3 exhibits oncogenic properties. Numerous clinical studies have reported frequent overexpression of MTA3 in tumor tissues, which is significantly associated with advanced p-TNM stage, lymph node metastasis, and poor prognosis (33, 34). MTA3 promotes NSCLC progression through several mechanisms. MTA3 knockdown induces G1/S phase arrest and downregulates the expression of cyclin A, cyclin D1, and phosphorylated Rb, thereby suppressing cell proliferation (33). Concurrently, MTA3 inhibits apoptosis by downregulating pro-apoptotic proteins, such as BAX and cleaved PARP, while upregulating the anti-apoptotic protein Bcl-2 (35). Additionally, MTA3 increases cell migration and invasion by interacting with HDAC2 (36). Moreover, MTA3 has been identified as a direct target of miR-495, forming a regulatory axis that plays a critical role in lung cancer growth and metastasis (37).

MTA3, functioning as an oncogene, is critically implicated in the pathogenesis of non-small cell lung cancer (NSCLC) and may have a potential association with anti-tumor immunity. The overexpression of MTA3 is hypothesized to modulate the expression of immune-related molecules on the tumor cell surface, thereby impacting immune cell recognition and cytotoxic activity. Specifically, MTA3 may regulate the expression of major histocompatibility complex (MHC) molecules, facilitating immune evasion by tumor cells (see **Table 1**, **Figure 3**). Additionally, MTA3 might influence cytokine secretion, contributing to the establishment of an immunosuppressive microenvironment that impedes the infiltration and activation of immune cells, consequently attenuating the anti-tumor immune response (see **Table 1**, **Figure 3**).

Currently, the majority of research concerning MTA3 in non-small cell lung cancer (NSCLC) is focused on preclinical exploration and early translational stages. Future investigations could utilize advanced technologies, such as gene editing and proteomics, to conduct an in-depth analysis of the transcriptional and post-transcriptional regulatory networks associated with MTA3 in NSCLC. Additionally, structural biology techniques may be employed to elucidate the molecular structural basis of interactions between MTA3 and proteins like HDAC, further identifying its downstream signaling pathways. Preclinical studies, including cellular and animal model experiments, could provide preliminary evaluations of the therapeutic potential of targeting MTA3 *in vivo*, as well as cautiously explore its potential clinical applications. Concurrently, clinical sample studies should be undertaken to assess the feasibility of employing MTA3 as a prognostic biomarker for NSCLC in preliminary clinical applications. Furthermore, the role of MTA3 within the tumor microenvironment and its involvement in immune regulation should be investigated to establish a foundational theoretical basis for its potential as a target in combination therapies. However, to facilitate the broad application of MTA3-related research findings in clinical practice, extensive rigorous and in-depth studies are still required for validation.

### 3.3 Hepatocellular carcinoma

HCC is a highly aggressive malignant tumor, and its pathogenesis is closely associated with multiple molecular alterations. MTA3 has emerged as a molecule of growing interest due to its aberrant expression and functional significance in HCC, with accumulating evidence highlighting its critical role in tumor progression. MTA3 is frequently overexpressed in the nuclei of HCC cells and is significantly associated with tumor metastasis and adverse patient prognosis (38). *In vivo* and *in vitro* functional studies demonstrated that MTA3 knockout significantly suppresses the proliferative, invasive, and metastatic potential of HCC cells (38). At the cellular level, siRNA-mediated silencing of MTA3 expression significantly reduces the *via*bility and migratory capacity of both HepG2 and Hepa1–6 cell lines (39), indicating that MTA3 plays a critical regulatory role in maintaining the malignant phenotype of HCC.

Mechanistic studies have elucidated the pathological significance of MTA3. Tunicamycin exerts antitumor effects by modulating the miR-22-3p/MTA3 axis (40), whereas the transient receptor potential channel TRPC1 may promote HCC progression by upregulating MTA3 and other associated genes (41), highlighting its central role in complex regulatory networks. Prior to clinical application, numerous critical issues remain in the research concerning MTA3. It is imperative to elucidate the regulatory mechanisms and functional distinctions associated with its nuclear/cytoplasmic localization switch in hepatocellular carcinoma (HCC) to gain a comprehensive understanding of its mechanism of action. A systematic analysis of the signaling networks in which MTA3 is involved is required, including the investigation of interactions between upstream and downstream molecules within the miR-22-3p/MTA3 axis and its downstream effector molecules, to construct a complete signaling map. Furthermore, there is a need to actively explore targeted strategies, such as the development of small molecule inhibitors or gene silencing therapies, and to assess their *in vivo* efficacy to identify novel therapeutic targets (see **Table 1**, **Figure 3**). Early translational research should also focus on a thorough evaluation of MTA3’s potential as a prognostic marker and therapeutic target to facilitate its clinical application.

### 3.4 Cervical and head-chest tumors

#### 3.4.1 Glioma

Glioma is the most common primary malignant tumor of the central nervous system. Accumulating evidence indicates that MTA3 plays a critical role in its pathogenesis. MTA3 is downregulated in glioma tissues, and its low expression is independently associated with higher WHO grades and unfavorable patient prognosis, indicating a potential tumor-suppressive function (42). Mechanistically, the long non-coding RNA HCG11 acts as a molecular sponge for miR-4425, thereby relieving miR-4425-mediated repression of MTA3 and subsequently inhibiting tumor growth (43). Conversely, layilin promotes tumor cell invasion by downregulating MTA3 and upregulating SNAI1 (44). The PARP inhibitor olaparib exhibits dose-dependent inhibition of glioma cell migration by upregulating MTA3 and E-cadherin, downregulating multiple pro-metastatic proteins, and modulating diverse signaling pathways, highlighting its potential as a therapeutic candidate for glioma treatment (72) (see **Table 1**, **Figure 3**). In forthcoming preclinical research, it is imperative to conduct an in-depth investigation into the regulatory network and molecular mechanisms of MTA3 in glioma. During the initial phases of translational development, a comprehensive evaluation of its potential as a prognostic biomarker and therapeutic target is essential. Such efforts aim to furnish novel insights and evidence that could significantly contribute to the prevention and treatment of glioma.

#### 3.4.2 Nasopharyngeal carcinoma

NPC is a malignant neoplasm arising from the mucosal epithelium of the nasopharynx. Its development, progression, and clinical outcomes are governed by several molecular mechanisms. A study using immunohistochemistry to assess MTA1, MTA2, and MTA3 expression in tissue microarrays from 152 patients with NPC demonstrated that MTA proteins are predominantly localized in the nucleus, and their associations with clinical parameters and prognosis exhibit ethnic variability. In the Han Chinese population, The expression of MTA3 demonstrated a positive correlation with female gender (r = 0.380, P = 0.011), suggesting that MTA3 expression levels were elevated in female patients with nasopharyngeal carcinoma compared to their male counterparts., while patients with high MTA1 expression exhibited poorer prognoses. Likewise, patients exhibiting elevated MTA3 expression tend to experience a less favorable prognosis. Contrarily, among individuals of Zhuang ethnicity, elevated MTA3 expression was significantly associated with advanced age, tumor recurrence, and metastasis, as well as independently predicted adverse outcomes (45). Several studies have confirmed that MTA family members exert pro-tumorigenic effects in NPC, consistent with previous findings (46, 73). Besides, MTA3-mediated biological functions demonstrate distinct ethnic-specific patterns (45). The correlation between MTA protein expression and nasopharyngeal carcinoma exhibits racial variability. This protein not only facilitates oncogenesis but also performs distinct biological functions, thereby serving as a potential biomarker for therapeutic interventions (see **Table 1**, **Figure 3**). Future research should prioritize preclinical and early translational studies to further elucidate these associations.

#### 3.4.3 Tongue squamous cell carcinoma

TSCC is a highly aggressive malignancy commonly occurring in the oral cavity. It is characterized by significant invasiveness and early metastatic potential. Accumulating evidence indicates that MTA3 expression is significantly downregulated in TSCC, and its reduced levels are significantly associated with tumor progression and poor prognosis (47–49) (see **Table 1**, **Figure 3**). MTA3 negatively regulates SOX2, a key transcription factor involved in cancer stem cell maintenance, thereby suppressing the stem-like properties and proliferative capacity of tumor cells (74). Analyses of animal models and clinical specimens demonstrated that the combination of low MTA3 expression and high SOX2 expression is correlated with poorer survival outcomes, indicating its potential utility as an independent prognostic biomarker (47) (see **Table 1**, **Figure 3**). Future research efforts should prioritize preclinical and early translational studies, particularly those examining transcriptional regulation and the interactions within stem cell pathways.

### 3.5 Tumors of the digestive system

#### 3.5.1 Esophageal cancer

MTA3 is highly expressed in the digestive system, particularly in the gastrointestinal mucosa, with the highest levels observed in the esophageal tissue. However, mRNA and protein expression of MTA3 were significantly downregulated in esophageal squamous cell carcinoma and esophagogastric adenocarcinoma, a finding consistently validated across multiple independent databases and clinical cohorts (50, 51). MTA3 forms a complex with the transcription factor GATA3, leading to the suppression of long non-coding RNA SOX2OT. This inhibition subsequently disrupts the SOX2OT/SOX2 signaling axis, effectively attenuating the proliferation and metastatic potential of esophageal cancer cells in both *in vitro* and *in vivo* models. Clinical survival analyses revealed that low MTA3 and high SOX2 expression are independently associated with poor patient prognosis (50) (see **Table 1**, **Figure 3**). In future research, it is imperative to investigate the preclinical and early translational dimensions, including transcriptional regulation and interactions with the tumor microenvironment.

#### 3.5.2 Gastroesophageal junction adenocarcinoma

GEJ adenocarcinoma is a highly aggressive malignancy characterized by early metastatic potential and poor prognosis. MTA3 functions as a key negative regulator of EMT, suppressing tumor invasion and metastasis through transcriptional repression of Snail, thereby preserving E-cadherin expression. Evidence indicates that MTA3 expression is significantly downregulated in GEJ adenocarcinoma tissues and highly metastatic cell lines, including OE-19 and FLO-1 (51). Immunohistochemical analysis of a cohort of 128 patients revealed that low MTA3 expression is significantly associated with adverse clinical outcomes and serves as an independent prognostic factor in multivariate Cox regression models. Further mechanistic investigations (51) have revealed that dysregulation of the MTA3/Snail/E-cadherin axis is closely associated with tumor differentiation status, lymph node metastasis, and advanced TNM staging. The combined assessment of these three markers improved the prognostic stratification accuracy, with a C-index of 0.78. Inactivation of this regulatory axis is associated with activation of EMT programs and increased metastatic capacity, consistent with previous studies (52, 53, 75) (see **Table 1**, **Figure 3**). Future research should aim to elucidate the epigenetic mechanisms governing MTA3 expression in GEJ adenocarcinoma, validate its clinical utility as a prognostic biomarker, and develop targeted therapeutic strategies for the MTA3/Snail/E-cadherin signaling pathway.

#### 3.5.3 Colorectal cancer

CRC is a prevalent malignant neoplasm of the gastrointestinal tract. MTA3 plays a significant role in CRC tumorigenesis and progression. In contrast to its tumor-suppressive function in breast cancer, MTA3 is frequently overexpressed in CRC and is significantly associated with advanced TNM stage, elevated Ki67 index, and poor prognosis (26). The oncogenic mechanisms of MTA3 in CRC involve activation of the Wnt/β-catenin signaling pathway, leading to increased expression of cyclin D1 and E and accelerated cell cycle progression. Additionally, MTA3 exerts anti-apoptotic effects by upregulating Bcl-2 and downregulating Bax, as well as improving resistance to 5-fluorouracil-based chemotherapy (26). Moreover, MTA3 promotes metastasis by modulating EMT. The long non-coding RNA PCAT1 promotes cancer cell invasion by regulating the MTA2/MTA3/Snail1/E-cadherin axis in conjunction with ZNF217 (54). Few studies have reported reduced MTA3 expression in certain highly metastatic CRC cell lines, with lower levels correlating with worse clinical outcomes (55), indicating a context-dependent dual role for MTA3 in CRC metastasis. Future research should concentrate on elucidating the background-dependent mechanisms governing its expression regulation and bidirectional effects. Additionally, the preclinical and early translational potential should be thoroughly investigated.

### 3.6 Genitourinary tumors

#### 3.6.1 Clear cell renal cell carcinoma

ccRCC is the most aggressive and malignant subtype of renal cancer, with the poorest prognosis among all renal tumor types. Emerging evidence indicates that miRNAs play key regulatory roles in ccRC pathogenesis and metastatic progression (76, 77). Ding D et al. (56) demonstrated that miR-367 significantly improved the migratory and invasive capabilities of ccRCC cells by directly targeting and suppressing MTA3 expression. Conversely, exogenous overexpression of MTA3 effectively reversed the miR-367-induced malignant phenotype (56), indicating that MTA3 inhibits tumor progression in ccRCC. Future research should prioritize investigating the downstream signaling pathways and epigenetic regulation of MTA3 in clear cell renal cell carcinoma (ccRCC) at both preclinical and early translational stages.

#### 3.6.2 Prostate cancer

Prostate cancer is an epithelial malignancy of the prostate gland. Integrative proteomic and pathway analyses have proven instrumental in elucidating the underlying molecular mechanisms (78). In a comparative study of matched prostate tumor tissues from African American and Caucasian American males, 45 proteins were identified that were significantly associated with cancerous transformation compared to adjacent non-malignant tissues, among which three were significantly downregulated. The MTA3-related signaling pathway was significantly enriched in tumor samples. Although there were no statistically significant differences in individual protein expression levels between African American and Caucasian American cohorts, differences in patterns of overexpression and pathway enrichment were detected. These findings indicate that dysregulation of the MTA3 pathway may contribute to prostate carcinogenesis and could provide novel insights for future research (57). Further validation of the functional mechanisms underlying this pathway is warranted (see **Table 1**, **Figure 3**).

### 3.7 Gynecological oncology

#### 3.7.1 Endometrial cancer

Endometrial cancer comprises a heterogeneous group of epithelial malignancies originating from the endometrium, the inner mucosal lining of the uterus. In this disease, MTA3 expression is closely linked to the ER signaling pathway and is modulated by estrogen through the ERα/ERβ system (58). Evidence indicates that MTA3 is frequently downregulated in endometrioid carcinoma, and its low expression correlates with increased metastatic potential. In contrast, non-endometrioid subtypes, including serous carcinoma, exhibit MTA3 overexpression. Elevated MTA3 levels in these aggressive variants are significantly associated with advanced surgical stage, lymph node metastasis, lymphovascular space invasion, and reduced progression-free and overall survival, establishing MTA3 as an independent prognostic factor (58, 59). Future research should focus on investigating the molecular mechanisms underlying the divergent MTA3 expression patterns across endometrial cancer subtypes. Given its prognostic relevance and association with key signaling pathways, MTA3 represents a promising biomarker for molecular subtyping and risk stratification (see **Table 1**, **Figure 3**). Future research should prioritize elucidating the molecular mechanisms responsible for their differential expression, conducting preclinical functional studies, and investigating their early translational potential as molecular subtypes, prognostic tools, and therapeutic targets.

#### 3.7.2 Choriocarcinoma

Choriocarcinoma is a highly malignant and aggressive trophoblastic tumor, characterized by high metastatic potential that poses significant challenges in clinical management. The role of MTA3 in regulating tumor invasion has attracted growing research interest. MTA3 is predominantly expressed in the nuclei of human trophoblastic and choriocarcinoma cells (60), demonstrating its involvement in promoting invasive behaviors by modulating cellular migration. Mechanistic studies have revealed that MTA3 functions in an ERβ-mediated signaling pathway. ERβ ligands, such as genistein, significantly downregulated MTA3 mRNA expression upon receptor activation. Reduced MTA3 levels subsequently alter downstream effectors, thereby upregulating mRNA expression of the transcriptional repressor Snail, and leading to increased protein expression of the intercellular adhesion molecule E-cadherin (61). This ERβ/MTA3/Snail/E-cadherin regulatory axis plays a critical role in suppressing the invasive capacity of JAR choriocarcinoma cells, as siRNA-mediated knockdown of ERβ completely reversed these molecular changes and abolished the inhibitory effect on cell invasion.

In summary, MTA3 is a key component of the MTA family and occupies a central position in the regulatory network governing invasiveness in choriocarcinoma (see **Table 1**, **Figure 3**). Future preclinical research should concentrate on elucidating the specific mechanisms of action of MTA3, as well as its synergistic interactions with related family members, to investigate its potential for early translational applications.

#### 3.7.3 Ovarian cancer

Ovarian cancer is characterized by insidious onset, rapid progression, and high propensity for invasion and metastasis, resulting in the highest mortality rate among all gynecological malignancies (79). MTA1 overexpression in metastatic ovarian cancer tissues is associated with downregulation of MTA3 and E-cadherin in tumor cells (62). Bruning et al. (59) reported significantly higher MTA3 expression levels in normal ovarian epithelial tissue compared to ovarian cancer specimens, implying that reduced MTA3 expression may contribute to tumorigenesis and disease progression. Evidence indicates that MTA3 suppresses β-hCG expression by binding to its transcriptional promoter region, thereby inhibiting its metastatic potential *in vitro*. Future preclinical research should concentrate on elucidating the downstream signaling pathways regulated by MTA3, investigate its potential utility as a prognostic biomarker, and develop early translational strategies aimed at restoring or mimicking its function.

### 3.8 Thymic epithelial tumors

MTA3 has been identified as a key regulator of the TME and immune modulation in TETs (63, 80, 81). Bioinformatics analysis revealed that MTA3 expression was significantly correlated with immune scores in TETs, where low expression levels were associated with impaired TME function, decreased cytotoxic activity, and poor patient prognosis. Further analyses revealed that MTA3 expression was positively correlated with the infiltration levels of multiple immune cell populations, including cytotoxic T cells, and co-expressed with corresponding immune markers (64). Gene set enrichment analysis revealed that MTA3 may significantly affect the TME by positively regulating multiple immune response pathways (64). These findings imply that MTA3 not only functions as a potential prognostic biomarker in TETs but also plays a critical role in modulating tumor immune surveillance by regulating immune cell infiltration and function. This regulatory role likely stems from MTA3’s function as a core component of the NuRD complex, participating in chromatin remodeling and transcriptional regulation of immune-related genes (see **Table 1**, **Figure 3**). Future studies should use clinical specimens and animal models to validate the specific molecular mechanisms underlying MTA3-mediated immune modulation in the TET microenvironment. Furthermore, investigating the relationship between MTA3 expression levels and response to immune checkpoint inhibitors may provide novel theoretical foundations and actionable targets for immunotherapy.

### 3.9 Leukemia

Leukemia is a group of malignant clonal disorders arising from hematopoietic stem or progenitor cells. Its hallmark features include aberrant proliferation and accumulation of leukemic cells in hematopoietic tissues, such as the bone marrow, infiltration into extramedullary organs and tissues, and suppression of normal hematopoiesis. MTA3, a cell type-specific subunit of the Mi-2/NuRD transcriptional corepressor complex, is co-expressed with the transcriptional repressor BCL-6 in germinal center B cells and plays a critical role in regulating B lymphocyte differentiation. BCL-6 suppresses terminal differentiation of B cells into plasma cells in an MTA3-dependent manner, and its repressive activity is modulated by acetylation status (18). Patients with high lipoprotein lipase mRNA expression in B-cell chronic lymphocytic leukemia (B-CLL) demonstrate significantly higher MTA3 signaling activity, which is associated with adverse cytogenetic profiles, shorter time to first treatment, and reduced overall survival (65). MTA3 knockdown impairs BCL-6-mediated transcriptional repression and disrupts normal B-cell gene expression programs. Conversely, ectopic expression of BCL-6 in plasma cells reverses cellular differentiation in an MTA3-dependent manner. By enhancing BCL-6 function, MTA3 promotes a dedifferentiated state in leukemic cells and contributes to disease progression, highlighting its potential as a prognostic biomarker and therapeutic target (see **Table 1**, **Figure 3**). Future research should prioritize elucidating the fundamental molecular mechanisms underlying the MTA3 signaling pathway, alongside other preclinical investigations. Additionally, efforts should be directed towards the exploration of small molecule inhibitors or antibody-based therapeutics, coupled with early translational assessments to evaluate their efficacy and safety.

### 3.10 Lymphoma

Lymphoma is a malignant neoplasm of the lymphoid hematopoietic system, characterized by uncontrolled lymphocyte proliferation, immune dysregulation, and multi-organ involvement (82) (see **Table 2**, **Figure 3**). MTA3 is highly expressed in B cells and physically interacts with the transcription factor BCL6, forming a functional complex that co-regulates key biological processes in germinal center B cells and contributes to lymphomagenesis (66). Evidence indicates that MTA3 is specifically overexpressed in germinal center B-cell-like diffuse large B-cell lymphoma, with expression levels positively correlated with cellular proliferation (66). BCL6 performs transcriptional regulation by recruiting different corepressor complexes, including SMRT/N-CoR and MTA3-containing NuRD complexes, enabling context-specific gene repression. This mechanistic insight provides a rationale for developing combination therapies targeting the BCL6 co-regulatory network (67). Moreover, MTA3 participates in regulating Tfh cell-associated gene expression programs, thereby modulating the tumor immune microenvironment (68). Future research should aim to elucidate the subtype-specific regulatory mechanisms of MTA3 across lymphoma entities, characterize the molecular basis of its interaction with BCL6, and evaluate the therapeutic potential of targeting the MTA3-BCL6 axis in precision oncology approaches (see **Table 1**, **Figure 3**).

**Table 2:** Expression of MTA3 in non-tumor diseases.

Disease	Mechanism pathway	Interacting genes or proteins	Expression	Functions	References
Hepatic fibrosis	EMT regulation (Snail family)	α-SMA,Col1A1,TβR, Bax,Bcl-2,Snail family	Downregulation	Regulation of HSC activation, ECM deposition and EMT	(27, 83–85)
Cardiac fibrosis	MTA3-p38 MAPK-E2F1 Axis	p38,MAPK,E2F1,α-SMA,CollagenI,TAK1,MKK4,JNK	Downregulation	Inhibit FMT and collagen deposition	(86–90)
IHPS	Association of the EML4-MTA3 gene locus	EML4	/	EML4-MTA3 is a new risk locus for IHPS	(91)
Stroke	The miR-138–GATAD2B–NuRD–MTA3–Wnt signaling axis modulates neural differentiation in dental pulp stem cells (DPSCs)	miR-138,GATAD2B,Wnt pathway molecules, DPSCs-related differentiation genes	Downregulation	Facilitate the differentiation of dental pulp stem cells (DPSCs) and promote neural tissue repair	(92)
Preeclampsia	The MTA3-NuRD complex regulates the invasion of EVT(MMP2/MMP9/Snail)	MMP2,MMP9,Snail,EVT-related genes	Downregulation	Inhibit the invasion of EVT; promote the occurrence of preeclampsia	(93–95)
IUGR	The MTA3-β-catenin axis regulates the proliferation of placental cells	β-catenin	Overexpression	Cell proliferation	(96, 97)
Disorders in testicular steroidogenesis	1. Abnormal production of testicular steroids 2. miR-146a-5p-MTA3 axis 3. Oxidative stress - NR4A1-MTA3 axis	NR4A1,miR-146a-5p	Downregulation	Maintain testosterone production and male reproductive capacity	(98–101)
Alzheimer’s disease	/	CHD5,Mi2 complex, Neurons Aging-related genes	/	Involved in neuronal gene regulation and aging-related pathways	(24)
Autism	/	PHB2,TNXB,DCTN2,RBM23	/	Participate in the regulation of autism-related signaling pathways	(102)
Sepsis	/	MAP1LC3A	/	It may affect the progression of sepsis through PPI	(103)

## 4 Expression of MTA3 in non-tumor diseases

### 4.1 Liver fibrosis

Liver fibrosis is a pathological consequence of chronic hepatic injury, characterized by excessive accumulation of extracellular matrix (ECM) components, and is primarily driven by the activation of hepatic stellate cells (HSCs). MTA3 plays a multifaceted inhibitory role in the development and progression of liver fibrosis through several molecular mechanisms. First, MTA3 epigenetically suppresses HSC activation by downregulating the expression of α-smooth muscle actin (α-SMA) and collagen type I alpha 1 chain, which are key markers of HSC transdifferentiation into myofibroblasts, thereby reducing ECM overproduction at its source (83, 84). Second, MTA3 promotes apoptosis in activated HSCs by modulating the expression of apoptotic regulators, specifically by upregulating pro-apoptotic Bax and downregulating anti-apoptotic Bcl-2, thereby overcoming the apoptosis resistance commonly observed in activated HSCs and limiting fibrotic expansion (85). Third, MTA3 negatively regulates EMT by directly interacting with Snail, a master transcription factor of EMT, preventing its binding to target gene promoters. Concurrently, MTA3 recruits the NuRD complex to repress Snail gene transcription, leading to reduced Snail protein levels. This dual mechanism helps preserve epithelial cell identity (as evidenced by sustained E-cadherin expression), suppresses mesenchymal transition, and indirectly mitigates liver fibrosis (27). Future research should prioritize elucidating the fundamental molecular mechanisms underlying the MTA3 signaling pathway, alongside other preclinical investigations. It should also explore the development of small molecule inhibitors or antibody-based therapeutics and undertake preliminary translational assessments to evaluate their efficacy and safety (see **Table 2**, **Figure 3**).

### 4.2 Cardiac fibrosis

Cardiac fibrosis is a prevalent pathological feature of various cardiovascular diseases, characterized by the transdifferentiation of cardiac fibroblasts into activated myofibroblasts and the excessive deposition of ECM proteins, particularly type I and type III collagen. These changes contribute to increased myocardial stiffness, impaired compliance, and progressive deterioration of cardiac function (86, 87). MTA3 plays a critical regulatory role in this process; its expression is significantly downregulated in fibrotic cardiac tissues, whereas experimental overexpression of MTA3 suppressed the expression of α-SMA and collagen I, thereby attenuating fibrotic remodeling and improving cardiac performance. This protective effect may be mediated by the modulation of the p38 MAPK-E2F1 signaling pathway (88). E2F1 functions as a pro-fibrotic transcription factor that represses MTA3 expression; however, emodin derivatives restored MTA3 levels by inhibiting E2F1 activity, consequently delaying cardiac fibrosis progression (89). Emerging evidence indicates that MTA3 may exert context-dependent effects on fibrotic processes, with some studies reporting that its overexpression can paradoxically promote fibrosis under specific conditions (90). Additionally, natural bioactive compounds, such as genistein, ameliorate cardiac fibrosis and improve cardiac function by activating the MTA3/TAK1/MKK4/JNK signaling cascade (104). Future preclinical investigations should focus on elucidating the bidirectional regulatory mechanisms of MTA3. Additionally, research should aim to develop strategies for drug development and early translational interventions targeting the associated signaling pathways.

### 4.3 Infantile hypertrophic pyloric stenosis

IHPS is a gastrointestinal disorder primarily affecting infants and young children. It is characterized by hypertrophy of the smooth muscle layer of the pyloric sphincter, leading to gastric outlet obstruction. Although several genetic loci have been associated with IHPS, they account for only a small proportion of disease risk. To identify additional susceptibility loci, Fadista J. et al. (91) performed a genome-wide meta-analysis involving 1,395 surgically confirmed cases and 4,438 controls, followed by independent validation in a replication cohort of 2,427 cases and 2,524 controls. Two novel risk loci, BARX1 and EML4-MTA3, were identified in this comprehensive genetic study, which were significantly associated with IHPS susceptibility (91). These findings provide important insights into the genetic architecture of IHPS and serve as a valuable foundation for future functional and mechanistic studies (see **Table 2**, **Figure 3**).

### 4.4 Stroke

Stroke is an acute cerebrovascular event characterized by the sudden rupture or occlusion of cerebral blood vessels, resulting in focal cerebral ischemia or hemorrhage and subsequent neuronal injuries. It is a major global health burden, marked by high incidence, mortality, disability, and recurrence rates. It is currently among the leading causes of long-term disability and death in adults worldwide (105). Zhou et al. (92) discovered in a murine model that transfection with miR-138 promotes degradation of the GATAD2B protein, destabilizes the NuRD complex, and induces nuclear-to-cytoplasmic translocation of MTA3. This cascade activates the Wnt signaling pathway, driving the differentiation of dental pulp stem cells into gamma-aminobutyric acid-ergic neurons. Consequently, this process facilitates structural reorganization and functional recovery of neural networks after a stroke and contributes to the repair of ischemic neuronal loss. These findings reveal a novel molecular mechanism underlying post-stroke neuro-regeneration and propose potential therapeutic targets for neural repair. Future studies should elucidate the clinical relevance of this pathway using human tissue samples, evaluate the safety, specificity (see **Table 2**, **Figure 3**), and delivery efficiency of miR-138-based vectors, and assess their therapeutic efficacy in diverse preclinical stroke models.

### 4.5 Preeclampsia

The invasion of extravillous trophoblast (EVT) cells is a critical process during early placentation, essential for the physiological remodeling of uterine spiral arteries and the establishment of adequate maternal-fetal circulation. Impaired EVT invasiveness is involved in the pathogenesis of several pregnancy-specific disorders, most notably preeclampsia (106, 107). Accumulating evidence indicates that MTA3 expression is significantly downregulated in placental tissues from patients with preeclampsia (93, 94). In MTA3-deficient trophoblast cells, key genes associated with invasive potential, such as matrix metalloproteinases-2 (MMP2) and MMP9, as well as the transcription factor Snail, are upregulated, indicating that MTA3 may act as a negative regulator of EVT invasion by repressing these pro-invasive factors (94). Moreover, as an integral component of the NuRD complex, MTA3 not only modulates EVT invasiveness and the expression of associated genes but also plays a critical role in the differentiation of cytotrophoblasts into syncytiotrophoblast and mature EVT lineages (21, 95). These findings highlight the multifaceted regulatory role of MTA3 in placental development. Future research should concentrate on elucidating the specific molecular mechanisms by which MTA3 regulates cellular invasion and differentiation (see **Table 2**, **Figure 3**). Additionally, further preclinical studies are warranted to assess its potential translational value as an early diagnostic marker or therapeutic target.

### 4.6 Intrauterine growth restriction

To investigate the molecular mechanisms underlying placental development in IUGR and the potential involvement of metastatic tumor antigen (MTA) family proteins, Alqaryyan et al. (96) established a dexamethasone-induced murine model of IUGR. They utilized quantitative real-time PCR, Western blotting, and immunohistochemistry to assess the expression levels of MTA1–3 in the basal and labyrinth zones of the placenta. Their results revealed increased MTA3 expression in the labyrinth zone of IUGR placentas, accompanied by reduced β-catenin levels and impaired proliferation. Alawadhi et al. (97) validated these findings and discovered that progesterone treatment normalizes dysregulated MTA1 and MTA3 expression, leading to improved placental function and pregnancy outcomes. These observations indicate a potential regulatory role for MTA proteins in placental pathophysiology associated with IUGR (see **Table 2**, **Figure 3**). Future studies should aim to elucidate the signaling pathways through which MTA3 modulates placental development and evaluate the translational potential of targeting the MTA family or implementing progesterone-based interventions for IUGR prevention and management.

### 4.7 Abnormal production of testicular steroids

MTA3, a multifunctional transcriptional co-regulator, is predominantly expressed in Leydig cells (LCs) and plays a critical role in regulating testicular steroidogenesis and spermatogenesis. Its expression is regulated at multiple levels: Insulin downregulates MTA3 in a concentration- and time-dependent manner; oxidative stress suppresses transcriptional activation through the nuclear receptor NR4A1, leading to reduced MTA3 expression, whereas NR4A1 overexpression counteracts this inhibition; miR-146a-5p directly targets the 3’ untranslated region of MTA3 to repress its expression, and this miRNA exhibits specific expression in LCs (98–100). Dysregulated MTA3 expression disrupts the balance of histone acetylation, characterized by decreased H4K5ac and H4K12ac and increased H3K9ac levels, thereby impairing spermatogenic progression. Environmental toxicants, such as arsenic exposure, induce male reproductive toxicity through mechanisms involving MTA3 downregulation, altered histone acetylation patterns, and disruption of intercellular junctions. Notably, exogenous MTA3 supplementation partially restored steroidogenic capacity and improved markers of male fertility in a murine model of diabetic testicular dysfunction (99, 101). Future research should systematically investigate the regulatory network linking MTA3 to key factors, such as NR4A1 and miR-146a-5p, to identify its downstream target genes in the steroid biosynthesis pathway (see **Table 2**, **Figure 3**). More research is required to elucidate the long-term effects and underlying molecular mechanisms by which environmental toxins (arsenic and heavy metals) and metabolic disorders (diabetes and obesity) modulate MTA3 expression. Given its central regulatory role, MTA3 is a promising therapeutic target, and researchers should focus on developing small-molecule agonists or miRNA antagonists to evaluate their potential for treating male infertility and hypogonadism.

### 4.8 Alzheimer’s disease

As a key component of CHD5, the rat brain-specific homolog, MTA3, integrates into the Mi2/NuRD chromatin remodeling complex and regulates gene expression through epigenetic mechanisms, potentially contributing to AD pathogenesis (24, 108). This complex modulates chromatin architecture, thereby influencing transcriptional programs governing neuron-specific, cell cycle-related, and aging-associated genes (109, 110). In the context of AD, MTA3 may contribute to neurodegenerative processes by regulating gene networks involved in synaptic function, neuronal survival, and β-amyloid metabolism (24). Functional impairment of MTA3 may lead to aberrant silencing of neuronal genes, inappropriate re-entry of post-mitotic neurons into the cell cycle, and compromised chromatin stability, all of which can exacerbate neuropathological alterations. Accordingly, MTA3 represents a pivotal molecular link between chromatin remodeling and AD pathogenesis. However, its precise mechanistic role requires further experimental validation (see **Table 2**, **Figure 3**).

### 4.9 Autism

Based on brain transcriptomic data analysis in individuals with autism spectrum disorder (ASD), MTA3 has been identified as a central regulatory gene within a co-expression network (102). As a component of the Mi2/NuRD chromatin remodeling complex, MTA3 is involved in regulating neuronal gene expression through epigenetic mechanisms, including histone modifications and chromatin reorganization. Dysregulation of MTA3 function may disrupt mRNA surveillance pathways and compromise calcium signaling homeostasis, leading to defective processing of synapse-associated mRNAs and diminished neuronal plasticity. These alterations contribute to the pathological mechanisms underlying synaptic developmental deficits and behavioral impairments in autism. Furthermore, MTA3 interacts with genes such as PHB2 and TNXB, indicating potential cooperative roles in mitochondrial function and ECM organization (102). While these findings highlight the putative involvement of MTA3 in ASD pathophysiology, the precise molecular mechanisms require further validation through functional experimental approaches (see **Table 2**, **Figure 3**).

### 4.10 Sepsis

Sepsis is a life-threatening condition characterized by organ dysfunction resulting from a dysregulated host response to infection. It represents the progression of infection-induced systemic inflammatory response syndrome to a pathological state marked by multiple organ failure (111). The precise role and underlying mechanisms of MTA3 in sepsis remain unclear. Recent bioinformatics analyses indicate that MTA3 may be involved in the pathogenesis of sepsis through PPI networks, particularly those involving genes such as MAP1LC3A (103). As a component of the Mi-2/NuRD chromatin remodeling complex, MTA3 can regulate the expression of genes associated with immune and inflammatory responses, cellular autophagy, and endothelial barrier integrity *via* epigenetic regulatory mechanisms, thereby contributing to sepsis development and progression. However, the specific molecular actions and functional relevance of MTA3 in this context require further investigation (see **Table 2**, **Figure 3**).

## 5 The mechanism of MTA3 in cancer and related pathologies: biological insights and translational perspectives

### 5.1 Clinical value of MTA3 in disease diagnosis and prognosis

#### 5.1.1 HCC

HCC is a prevalent and invasive malignant tumor, and its progression is driven by complex molecular networks in which MTA3 has emerged as a key player. Recent studies have revealed that MTA3 functions not only as an independent prognostic biomarker in HCC but also as a key oncogenic factor involved in tumor progression and immune modulation, highlighting its significant potential for clinical translation (39). Clinical cohort analyses have elucidated the robust prognostic value of MTA3. Evidence indicates that MTA3 expression is significantly upregulated in HCC tissues compared to adjacent non-tumor tissues, and its overexpression is strongly associated with reduced overall survival. Multivariate Cox regression analyses confirmed that MTA3 is an independent prognostic risk factor, retaining significance after adjusting for conventional clinical parameters, including TNM stage and histological grade. Moreover, predictive models incorporating MTA3 expression exhibited high discriminatory accuracy, positioning MTA3 as a reliable biomarker for risk stratification and prognostic evaluation in patients with HCC.

The oncogenic function of MTA3 is mediated through a finely tuned multi-tiered regulatory network. Upstream, the circular RNA circ_0021093 acts as a competing endogenous RNA by competitively binding miR-766-3p, thereby sequestering this miRNA and relieving its repression of MTA3 mRNA, leading to upregulation of MTA3 expression. This enhances the proliferation, migration, and invasion of HCC cells while suppressing apoptosis (118). Downstream, MTA3 functions as an epigenetic regulator that directly drives malignant phenotypes through various mechanisms, such as histone modification. MTA3 plays a key role in shaping an immunosuppressive TME. Its expression is positively correlated with immune checkpoint molecules, including PD-L1, which promotes the recruitment of inhibitory immune cells, such as regulatory T cells, and impairs the antitumor activity of effector lymphocytes, particularly CD8+ T cells, thereby facilitating immune evasion. Experimental evidence has confirmed that MTA3 knockdown effectively suppresses the malignant behaviors of HCC cells and partially reverses immunosuppressive conditions within the TME (39).

Researchers have identified jaspamycin, a marine-derived small-molecule compound, as a candidate for targeting the oncogenic function of MTA3. This compound specifically binds to MTA3 and downregulates its expression. *In vitro* experiments revealed that jaspamycin significantly reduced HCC cell *via*bility, induced cell cycle arrest, and reversed the immunosuppressive tumor phenotype. Molecular docking studies have confirmed its high-affinity interaction with MTA3, making it a promising candidate for therapeutic development and a structural foundation for designing precision therapies targeting MTA3 (39) (see **Table 3**).

**Table 3:** Clinical applications of MTA3 in various diseases (diagnosis and treatment).

Classification	Disease	Mechanism pathway	Interacting genes or proteins	Expression	Functions	References
Biomarker	HCC	1.miR-22-3p-MTA3 axis 2.circ_0021093/miR-766-3p/MTA3 axis	miR-22-3p,circ_0021093,miR-766-3p,TRPC1	Nuclear Overexpression cytoplasmic Downregulation	High expression of MTA3 in the nucleus is associated with metastasis and poor prognosis; it is an independent biomarker for the prognosis assessment of HCC	(39–41, 103)
	GEJ	EMT (Snail/E-cadherin)	Snail, E-cadherin	Downregulation	Associated with metastatic potential and poor prognosis, it serves as an independent prognostic indicator	(51–53)
	Colorectal cancer	1.Activation of the Wnt signaling pathway (Cyclin D1/Cyclin E) 2.EMT (Snail1/E-cadherin) 3.PCAT1/MTA2/MTA3/Snail1 axis	Wnt target protein, Cyclin D1, CyclinE, Snail1, E-cadherin, PCAT1, MTA2	Overexpression	It is positively correlated with advanced TNM staging and high Ki67 index; negative expression of MTA3 is an independent poor prognostic marker	(54, 55)
Therapy	Pancreatic cancer	1.NF-κB signaling pathway 2.STAT3 signaling pathway	CRIP2,NF-κB/p65	Overexpression	It can enhance the resistance of pancreatic ductal adenocarcinoma cells to gemcitabine	(112, 113)
	Cardiac fibrosis	MTA3-p38 MAPK-E2F1 axis (regulating FMT)	p38MAPK,E2F1,α-SMA,CollagenI,TAK1,MKK4	Downregulation	Drug-mediated overexpression of MTA3 can inhibit cardiac fibrosis	(89, 114–117)

In summary, MTA3 is regulated by upstream circRNA/miRNA networks and contributes to HCC progression and immune evasion through epigenetic mechanisms. Its expression level has been identified as an independent prognostic indicator. Future research should elucidate the specific epigenetic pathways through which MTA3 modulates immune checkpoint expression, with particular emphasis on evaluating the synergistic potential of MTA3-targeted agents, such as jaspamycin, in combination with existing immunotherapies. Such investigations will facilitate clinical translation and may lead to novel mechanism-based therapeutic strategies for patients with HCC.

#### 5.1.2 Adenocarcinoma of the gastroesophageal junction

AEG is a malignant neoplasm originating at the anatomical interface between the esophagus and stomach, with its global incidence increasing steadily in recent years (119). The current standard of care for AEG primarily involves surgical resection, combined with perioperative chemotherapy, radiotherapy, and targeted therapies. However, due to the tumor’s high invasiveness and propensity for early lymph node metastasis, patients often experience high rates of postoperative recurrence and poor overall prognosis. The conventional TNM staging system has limitations in providing precise risk stratification, highlighting an urgent need for reliable molecular biomarkers to identify high-risk individuals and guide personalized therapeutic strategies (120, 121). Recent evidence (51) indicates that MTA3 plays a significant role in the progression and prognostic assessment of AEG. Elevated MTA3 expression is associated with favorable clinicopathological characteristics, including well-to-moderately differentiated histology, earlier disease stages (I/II), and absence of lymph node metastasis. Furthermore, MTA3 expression was significantly positively correlated with the epithelial marker E-cadherin (r = 0.629, *P* < 0.001). The co-expression patterns of MTA3, Snail, and E-cadherin demonstrated substantial clinical relevance. The MTA3-negative/Snail-positive/E-cadherin-negative phenotype was significantly associated with lymph node metastasis, advanced disease stage, and poorly differentiated tumors. In a cohort study of 128 patients with AEG, those with MTA3-positive tumors demonstrated significantly improved overall survival compared to those with MTA3-negative tumors (*P* < 0.001), with this survival advantage consistently observed across both lymph node metastasis-positive and lymph node metastasis-negative subgroups. Multivariate Cox regression analysis further confirmed that MTA3 expression (hazard ratio = 0.442, *P* = 0.001) serves as an independent prognostic factor, independent of established variables, such as lymph node status and gender (51) (see **Table 3**).

In summary, MTA3 is not only involved in regulating EMT and tumor metastasis but also functions as a reliable independent prognostic biomarker in AEG, facilitating the identification of high-risk patient populations. Future research should investigate the molecular mechanisms underlying MTA3 involvement in AEG pathogenesis, assess its potential as a therapeutic target, and validate its clinical translational value through multicenter prospective studies. Such efforts are essential for advancing precision medicine strategies for patients with AEG.

#### 5.1.3 CRC

CRC is a malignant neoplasm arising from the mucosal epithelium of the colon or rectum. As one of the most prevalent gastrointestinal malignancies, CRC remains challenging in clinical management despite current multimodal treatment strategies centered on surgical resection, primarily due to the high rates of postoperative recurrence and suboptimal accuracy in prognostic assessment (122, 123). Conventional clinical and pathological parameters exhibit limitations in predicting patient outcomes, underscoring the need for more reliable molecular biomarkers to improve risk stratification and guide therapeutic decision-making (124). Evidence indicates that MTA3 expression serves as an independent predictor of recurrence and overall survival in CRC (55). Loss of MTA3 expression is associated with an increased risk of recurrence and shorter overall survival. In a survival analysis of 178 patients with CRC, the median overall survival was only 36.0 months in the MTA3-negative group, whereas it was not achieved in the MTA3-positive group (*P* = 0.011). Multivariate analysis confirmed that MTA3 expression independently affects prognosis, with predictive power comparable to established factors, including tumor differentiation grade, depth of invasion, lymph node metastasis, and TNM stage. Its prognostic value remains significant after adjusting for potential confounders, including gender, age, and tumor location (55) (see **Table 3**).

In summary, MTA3 is a strong and independent molecular biomarker for identifying subgroups at high risk of recurrence and poor outcomes in CRC, demonstrating significant potential for clinical translation. Future studies should investigate the mechanistic roles of MTA3 in CRC and validate its prognostic utility in multicenter, prospective cohort studies to facilitate its integration into clinical practice and the development of personalized treatment strategies.

### 5.2 Application of MTA3 in disease treatment

#### 5.2.1 Pancreatic ductal adenocarcinoma

**Figure 4:**
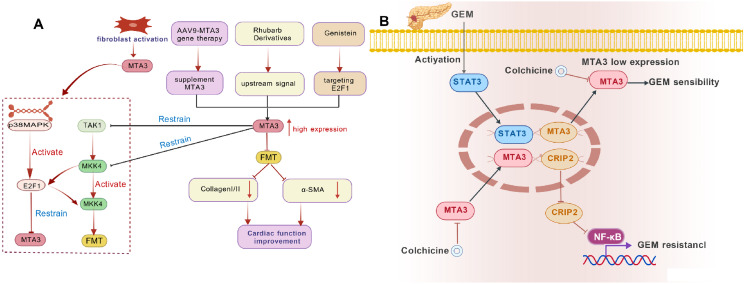
Relevant signal pathways of gemcitabine in pancreatic cancer. **(A)** illustrates the mechanism of action of the drug in cardiac fibrosis, and **(B)** shows the mechanism of action of GEM in PDAC.

PDAC is the most lethal gastrointestinal malignancy, with a five-year survival rate of 10% (125). Approximately 80% of patients are deemed ineligible for surgical resection due to rapid tumor progression and early metastasis (126). In advanced stages, survival extension primarily depends on palliative therapies, such as adjuvant chemotherapy (112). Gemcitabine (GEM) is the cornerstone of standard chemotherapeutic regimens. However, GEM resistance is one of the primary causes of treatment failure in PDAC, making resistance elimination and drug sensitivity improvement critical challenges in therapeutic management (113). CRISPR library screening revealed a significant enrichment of MTA3 in GEM-treated surviving PDAC cells. Bioinformatics and histological evaluations confirmed a strong association between MTA3 expression and GEM resistance (127). *In vitro* and *in vivo* experiments revealed that MTA3 is a key mediator of GEM resistance in PDAC, increasing cellular resistance to GEM and positioning it as a promising therapeutic target for combination therapy (127). As a component of the Mi-2/NuRD transcriptional repressor complex, MTA3 activates the NF-κB signaling pathway by suppressing CRIP2, a transcriptional repressor of NF-κB/p65, thereby promoting GEM resistance (see **Figure 4**). Furthermore, GEM treatment activates the STAT3 signaling pathway, leading to the upregulation of MTA3 expression in PDAC cells and contributing to acquired resistance (127). In animal models, colchicine suppresses MTA3 expression and increases tumor cell sensitivity to GEM (127) (see **Table 3**).

Future research should elucidate the molecular interactions between MTA3 and the NF-κB and STAT3 signaling pathways to identify key regulatory nodes. Concurrently, systematic investigation of alternative pharmacological agents capable of inhibiting MTA3 expression or disrupting its resistance-mediated signaling pathways is warranted. Large-scale clinical trials are required to evaluate the efficacy and safety of MTA3-targeted combination chemotherapy regimens, facilitating the development of more effective treatment strategies for patients with PDAC.

#### 5.2.2 Cardiac fibrosis

Cardiac fibrosis is characterized by excessive proliferation of cardiac fibroblasts and aberrant collagen deposition, leading to myocardial tissue stiffening and impaired cardiac function (86). Currently, no disease-specific anti-fibrotic therapies are available for cardiac fibrosis. Conventional treatments, such as angiotensin-converting enzyme inhibitors, angiotensin II receptor blockers, and aldosterone antagonists, can only slow fibrosis progression but are unable to reverse established scar tissue (114, 128–130). By modulating the critical fibroblast-to-myofibroblast transition pathway, MTA3 emerges as a novel therapeutic target for treating cardiac fibrosis. Developing small-molecule modulators targeting the MTA3-p38 MAPK-E2F1 signaling axis, such as p38 MAPK inhibitors or E2F1 antagonists, could be a promising strategy for reversing fibrotic remodeling. For example, genistein improves cardiac dysfunction induced by mechanical overload and attenuates cardiac fibrosis by regulating the MTA3/TAK1/MKK4/JNK signaling cascade, highlighting its potential as a novel agent for preventing and treating fibrosis-related cardiovascular diseases (104) (see **Table 3**). Additionally, emodin derivatives exert anti-fibrotic effects by targeting E2F1, restoring MTA3 expression, and disrupting the pro-fibrotic cascade. This mechanism offers significant promise for developing clinical therapeutics for cardiac fibrosis (89, 115, 116). Studies using adeno-associated virus serotype 9 vectors to achieve MTA3 overexpression have demonstrated significant therapeutic efficacy in animal models, highlighting the potential of MTA3-targeted gene therapy and paving the way for future clinical translation (131) (see **Figure 4**). Future clinical trials should evaluate the safety and efficacy of MTA3 modulators in fibrosis-associated conditions, including myocardial infarction and heart failure. Rational design of small-molecule inhibitors or activators targeting the MTA3 signaling pathway, as well as the development of gene therapy vectors specific for MTA3, warrants further investigation.

## 6 Conclusions and future perspectives

As the central component of the nucleosome remodeling and deacetylase complex (NuRD), MTA3 plays a pivotal role in essential biological processes, including chromatin remodeling, regulation of gene transcription, and protein-protein interactions, facilitated by its diverse functional domains. It exhibits intricate and significant biological functions across a range of cancers and non-neoplastic diseases, such as liver fibrosis.

In various cancer types, MTA3 demonstrates a distinct dual functionality. It may serve as a tumor suppressor, curbing the excessive proliferation and metastasis of tumors, as observed in breast cancer; conversely, it can also operate as a tumor promoter, facilitating cancer progression and exacerbation, as exemplified in non-small cell lung cancer (NSCLC). This ostensibly paradoxical behavior is primarily contingent upon several factors, including the cancer type, cellular milieu, and molecular context. Tumor types exhibit considerable diversity in their biological characteristics, molecular mechanisms, and microenvironments. The disparate origins of tumor cells, differences in gene expression profiles, and the activation states of signaling pathways can all contribute to MTA3 exhibiting varied functions and mechanisms of action across different tumors. Furthermore, specific gene mutations may modify the interaction of MTA3 with other molecules, thereby resulting in its diametrically opposed roles in distinct tumors.

The expression and function of MTA3 are meticulously regulated by a multitude of factors, including transcription factors, epigenetic modifications, and non-coding RNAs, which together constitute a complex regulatory network. This sophisticated network facilitates MTA3’s ability to display distinct functional patterns across various diseases, cell types, and stages of disease progression. For example, in colorectal cancer, MTA3 functions as an anti-apoptotic factor by upregulating Bcl-2 and downregulating Bax, thereby contributing to the malignant progression of tumors. Conversely, in models of liver fibrosis, MTA3 exhibits a pro-apoptotic effect, a mechanism intricately linked to the cellular activation state, tissue-specific regulation of signaling pathways, and variations in the microenvironment and epigenetic modifications. In the early stages of fibrosis, the pro-apoptotic activity of MTA3 can eliminate activated hepatic stellate cells (HSCs) and impede the progression of fibrosis. However, in advanced liver cirrhosis, the expression of MTA3 may diminish, resulting in inadequate apoptosis and exacerbating tissue hardening.

The expression level of MTA3 in various diseases is significantly associated with clinical pathological characteristics and prognosis, highlighting its substantial potential as a biomarker for disease diagnosis and prognosis. Concurrently, due to its pivotal role in disease pathogenesis and progression, MTA3 is considered a highly promising therapeutic target. Therapeutic strategies aimed at MTA3, such as employing PROTAC technology to degrade MTA3 in tumor treatment to enhance chemotherapy sensitivity, and modulating MTA3 expression (e.g., utilizing miR-32 inhibitors) to reverse fibrosis in liver conditions, are anticipated to offer novel pathways for disease treatment.

### 6.1 Future prospects

Currently, while MTA3 has made significant advancements in disease research, it remains largely in the preclinical and early translational discovery phases. Consequently, substantial progress is required before it can be effectively applied in clinical settings. Future research endeavors should concentrate on several key areas to enhance our understanding and facilitate the translation of MTA3-related research into clinical applications. At the mechanistic level, it is imperative to employ multi-omics integration analysis methods, utilizing extensive clinical samples and suitable animal models, to thoroughly investigate the specific mechanisms by which MTA3 influences disease onset and progression. Particular attention should be given to elucidating the detailed mechanisms of MTA3 function at the cellular and molecular levels, including its involvement with cancer stem cells, interactions with non-coding RNAs, regulation through post-translational modifications, and its specific role within the tumor microenvironment. For example, utilizing single-cell sequencing and spatial transcriptomics methodologies allows for the analysis of the dynamic regulatory network of MTA3 within specific cell subpopulations, thereby offering a more precise theoretical foundation for targeted therapeutic interventions.

Regarding the optimization of experimental models, given the existing disparities among current models in replicating the onset and progression of human diseases, it is imperative to refine the selection and design of these models. Integrating multiple experimental models can enhance the reliability and reproducibility of research findings. In the context of tumor research, for instance, alongside traditional cell-based experiments and animal models, the incorporation of organoid models—which more closely mimic the physiological environment of the human body—can facilitate a more accurate simulation of the complex characteristics of tumors.

To standardize detection methodologies, it is imperative to develop uniform experimental protocols and implement robust quality control systems. This includes harmonizing detection techniques to mitigate the influence of methodological discrepancies on research outcomes. Concurrently, it is essential to enhance the comparison and validation of diverse detection methods to ensure the precision and comparability of research findings. Such standardization is vital for the accurate assessment of MTA3’s expression levels, protein activity, and molecular interactions, thereby providing reliable data to support future clinical applications.

In the pursuit of developing targeted therapeutic strategies, it is imperative to conduct comprehensive research into the composition and dynamic regulatory mechanisms of the MTA3-NuRD complex. This involves elucidating the specific role of each subunit within the complex to inform the proposal of innovative targeted therapeutic approaches. Concurrently, it is essential to engage in drug development research targeting MTA3, which includes the screening and design of small molecule agonists or antagonists characterized by high efficacy and low toxicity. Additionally, the development of regulatory drugs based on non-coding RNA should be pursued to furnish more effective modalities for disease treatment.

By conducting comprehensive research across the aforementioned dimensions, it is anticipated that the full mechanism of MTA3 in the pathogenesis and progression of diseases will be progressively elucidated. This endeavor aims to establish a robust foundation for its clinical application as a diagnostic marker and therapeutic target. Such advancements are expected to propel MTA3-related research from the preclinical and early translational discovery phases to well-established clinical applications, ultimately offering new prospects for enhancing patient health outcomes and prognoses.
